# Enucleation of Chronic, Painful, and Irreparable Traumatic Injured Eyeball in a Local‐Breed Ox and Its Outcome

**DOI:** 10.1002/ccr3.72586

**Published:** 2026-04-21

**Authors:** Getachew Tadesse

**Affiliations:** ^1^ Department of Veterinary Medicine Woldia University Woldia Ethiopia

**Keywords:** enucleation, injured eye, ox, tarsorrhaphy, transpalpebral

## Abstract

An 8‐year‐old local‐breed ox presented with a severe, purulent, and malodorous injury to the right eye. The ox was restrained in a standing position, and then the site was prepared aseptically. A Peterson nerve block was applied using 2% lidocaine. The surgical technique involved a transpalpebral elliptical incision, removal of the globe, orbital packing with gauze, and skin closure using an interlocking silk suture pattern. Postoperative care included a 3‐day course of meloxicam and oxytetracycline, regular wound care, and orbital packing changes. Sutures were removed on Day 14, leading to an uncomplicated recovery.

## Key Clinical Message

This case report demonstrates detailed surgical procedures and techniques of transpalpebral enucleation for irreparable bovine ocular injuries, and it confirms an effective and humane surgical solution to ensuring pain relief, infection control, and full recovery with proper aftercare.

## Graphical abstract

Transpalpebral enucleation for an irreparable ocular injury in an ox. Clinical presentation of the damaged right eye. Preparation of the site, Peterson nerve block, and temporary tarsorrhaphy. Circumferential elliptical skin incision and excision of the damaged eye (enucleation). Orbital packing followed by interlocking silk sutures. Postoperative follow‐up and successful recovery.

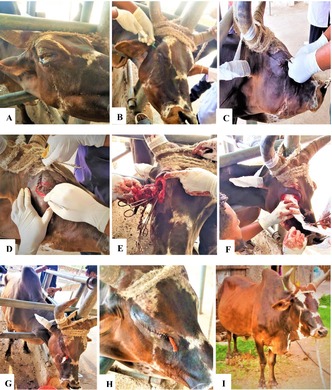

## 1 Introduction

The eyes are visual organs located in the bony orbits of the skull. It is composed of the conjunctiva, eyelids, lacrimal apparatus, extraocular muscles, and eye chambers that hold refractive media [1]. Injuries of the eye, such as scratches, abrasions, contusions, and punctures (penetrating), are prevalent in cattle. The causes of these injuries are mishaps, chemical exposure, or foreign objects. Treatment of ocular injuries may involve surgery and medication [2].

The surgical techniques that are commonly used to treat eye conditions and damage are evisceration, enucleation, and exenteration [3]. Evisceration removes intraocular contents while preserving the scleral shell and extraocular muscles, whereas enucleation involves total excision of the globe and optic nerve. Conversely, exenteration is the radical evacuation of all orbital contents, including the globe, adnexa, and periorbital tissues [2, 3]. Depending on the situation, these techniques can result in acceptable cosmetic outcomes and alleviate pain and suffering [4]. Enucleation is one of the most frequent surgeries done on cattle to treat infections and irreparable eye damage [5]. The approaches to eye enucleation are transpalpebral or transconjunctival. The transpalpebral approach is considered ideal for infected and neoplastic tissue to prevent orbital contamination [6].

Enucleation is a decisive surgical intervention in bovines for severe, irreversible ocular conditions such as neoplasia, penetrating trauma, and persistent end‐stage infections. These pathologies often cause considerable and refractory pain, with no potential for functional recovery [2, 7]. The objective of this report was to describe in detail the successful application of enucleation as a definitive treatment for a chronic, painful, and infected eye in a local‐breed ox.

## 2 Case Description and Management Procedures

### 2.1 History

An 8‐year‐old local breed ox was presented to Professor Feseha Geberab Memorial Veterinary Teaching Hospital, College of Veterinary Medicine and Agriculture, Addis Ababa University, with a chronic right eye injury. The history revealed the trauma was caused by an impact with a stick 2 weeks earlier. Initial treatment failed to resolve the condition, which subsequently worsened with the onset of suppurative, foul‐smelling exudate.

### 2.2 Clinical Findings and Physical Examination

**FIGURE 1:**
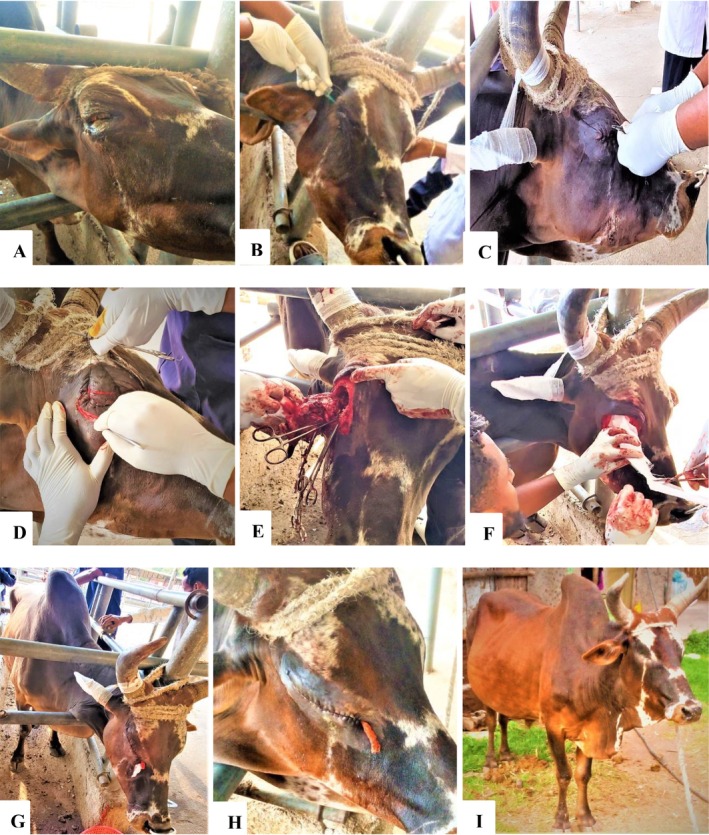
Enucleation of an injured eye and its outcome in a local‐breed ox. (A) Preoperative presentation of the injured right eye. (B) Administration of a Peterson nerve block. (C) Tarsorrhaphy (eyelid closure). (D) Initial elliptical skin incision. (E) Removal of the globe, nictitating membrane, and eyelid margins. (F) Orbital packing with sterile gauze. (G) External placement of the gauze tip at the medial commissure. (H) Healing progress on postoperative day 3. (I) Outcome.

Upon a thorough physical examination, the right eyeball was damaged and severely degenerated with continuous purulent lacrimal secretion (Figure 1A), which had a foul smell. Moreover, the ox was reactive and showed pain upon palpation of the affected eye. The heart rate was 72 beats per minute, the rectal temperature was 38°C, the respiratory rate was 24 breaths per minute, and the visible mucous membranes were pink. Based on anamnesis and clinical examination, the ox was diagnosed with an irreparable, chronic, and painful injury to the right eye. Therefore, the ox was admitted for surgical enucleation.

### 2.3 Preoperative Preparation

Oxytetracycline (10%, 0.1 mL/kg im; Shenyang Sunvictor Pharmaceutical Co. Ltd., China) was given prior to surgery in order to reduce the risk of infection. The right ear was dressed with gauze and secured to the horn to maintain access to the surgical site (Figure 1C). Following the induction of local anesthesia, the periorbital region was cleansed with water and soap. The eyelashes were clipped, and the surrounding hair was clipped and shaved. The surgical field was then aseptically prepared by scrubbing with a solution of Savlon (cetrimide 3% and chlorhexidine gluconate 0.3%), followed by a final application of iodine tincture.

### 2.4 Anesthesia and Restraint of the Ox

The ox was restrained in a standing position within a cattle crush using ropes and a bull holder. Regional anesthesia was achieved via a Peterson nerve block (Figure 1B), performed according to the standard technique [8]. Using lidocaine hydrochloride (2%, Mackur Laboratories Limited, India), the procedure began with the subcutaneous infiltration of 3 mL to desensitize the skin over the injection site. The needle was then advanced through the bony notch formed by the supraorbital process and the zygomatic arch, directed toward the orbitorotundum foramen. Upon reaching the foramen, the needle was slightly withdrawn, negative aspiration was confirmed, and 10 mL of lidocaine was deposited. Finally, a line block was administered by infiltrating 0.5–1 mL of lidocaine per 1–2 cm along the planned eyelid incision line.

### 2.5 Surgical Site and Approach

The transpalpebral approach was selected, with a circumferential elliptical skin incision placed around the eyelid margins (Figure 1D).

### 2.6 Surgical Procedures

Initially, the eyelid was temporarily closed (tarsorrhaphy) with a simple continuous suture pattern using nylon size 3 (Figure 1C) to facilitate a cleaner elliptical incision and handling of the surgical field. Then, a sharp elliptical skin incision was made approximately 1–2 cm from the palpebral margin using the inner contour of the bony orbit as a guide (Figure 1D). Hemorrhage during operation was controlled by gauze pressure, hemostatic forceps, and ligation. The eyeball was carefully freed from its attachments by dissection and cutting of the subcutaneous tissue, fascia, and the orbicularis oculi muscle using a scalpel and blunt scissors. The orbital apex was fully exposed by sharply cutting the medial and lateral canthal ligaments.

The optic nerve and its associated vasculature were isolated and clamped as far caudally as possible using two curved hemostats. Then, cut the optic nerve and completely remove the globe, nictitating membrane, and attached eyelid margins (Figure 1E). The orbit was packed with sterile gauze to arrest hemostasis (Figure 1F) and then irrigated with sterile saline to remove the remaining clot. Lastly, sterile gauze was packed and placed in the orbit. To make removal easier, the packed gauze's end tip remained outside at the medial commissure (Figure 1G).

Subcutaneous tissue was closed with a simple continuous pattern by using Vicryl size 2‐0, whereas skin was closed with a continuous interlocking pattern by using silk size 2 (Figure 1H). During closure, a small opening was preserved at the medial commissure for changing and removal of packed gauze. Finally, the site was cleansed with iodine tincture, and the patient was discharged.

### 2.7 Postoperative Care

The ox was confined to a shaded enclosure during the initial recovery period. Administration of oxytetracycline (10%, 0.1 mL/kg im; Shenyang Sunvictor Pharmaceutical Co. Ltd., China) was continued up to 4 days. Additionally, meloxicam (0.5 mg/kg, im, Ashish Life Science Pvt. Ltd., Mumbai, India) was given for 3 days as an anti‐inflammatory and analgesic. The surgical site was cleansed regularly every other day with iodine tincture until the wound healed. The sterile‐packed gauze was changed every other day. The size of the gauze was gradually reduced to promote healing from the depth of the orbit. The sutures were removed on the 14th day.

## 3 Outcome and Discussion

The ox recovered well (Figure 1I), except for intermittent blood‐tinged mucoid nasal discharge on the second and third days. The discharge subsided spontaneously, and it is likely related to the surgical manipulation near the nasolacrimal duct or sinus communication, a known potential sequela of deep orbital procedures. Similar to the current case, Schulz and Anderson [5] and Thiry et al. [7] justified enucleation as a standard and definitive surgical solution for serious ocular injuries, infection, or neoplasia when medical treatment is ineffective, and animal well‐being is jeopardized by persistent pain. The transpalpebral approach, which was used in this case, is especially advised when infection is present because it reduces the possibility of intraoperative orbital contamination and helps to eliminate fully infected tissues [2, 6].

This case reinforces that enucleation is a viable and effective procedure in cattle, with minimal long‐term impact on the animal's overall health or production capabilities. The successful outcome underscores the importance of a systematic approach encompassing accurate diagnosis, precise surgical technique, and diligent postoperative care in managing severe ocular conditions in production animals.

## 4 Conclusion

The report describes the successful surgical management of a chronic, painful, and infected eye injury in an ox through enucleation via a transpalpebral approach. The procedure effectively alleviated the animal's pain, eliminated the source of infection, and prevented further systemic complications. The ox made a complete recovery, demonstrating the procedure's efficacy and its negligible impact on the animal's long‐term well‐being. The case highlights enucleation as a crucial and humane surgical intervention in bovine medicine for irreparable ocular conditions, emphasizing that thorough pre‐operative preparation, aseptic technique, appropriate anesthesia, and consistent postoperative care are fundamental to achieving optimal outcomes.

## Author Contributions

**Getachew Tadesse:** conceptualization, data curation, investigation, methodology, resources, software, supervision, validation, visualization, writing – original draft, writing – review and editing.

## Funding

The author has nothing to report.

## Ethics Statement

In accordance with the institutional policies of Addis Ababa University, College of Veterinary Medicine and Agriculture, formal approval from an animal ethics committee was not required for a case report.

## Consent

The informed verbal and written consent was obtained from the animal's owner for the procedure and for the use of clinical data and images for scientific publication.

## Conflicts of Interest

The author declares no conflicts of interest.

## Data Availability

Since this is a case report no data was recorded.
